# On Rheumatism, Rheumatic Gout, or Sciatica, Their Pathology, Symptoms and Treatment

**Published:** 1854-06

**Authors:** 


ART. III.—*On Rheumatism, Rheumatic Gout, and Sciatica, their Pathology, Symptoms, and Treatment*, by HENRY FULLER, M. D., Contab., Fellow of the Royal College of Physicians, London; assistant Physician to St. George's Hospital, &c., &c.

SYDENHAM styled rheumatism "the poor man's gout." The disease of the poor man has attained in our day a wonderful ascendency over its patrician congener. Gout is a disorder now seldom spoken of, hardly dreaded at all, a stranger to most young practitioners in our country. It seems to have been superseded by rheumatism, which has proved to be one of the most formidable of diseases. Rheumatism is not only a disease of great frequency and of extreme suffering, but is now known to entail other diseases of the most terrible character. Affections of the heart, it is now admitted, result more frequently from rheumatism than from any other cause. These affections often complicate and render formidable the mildest cases of rheumatism, not only inducing present distress but darkening all the future of the sufferer. Their incursion moreover may be so insidious as to escape the detection of the physician, and thus they often pass to a point where remedies are unavailing before the patient is aware of his danger.

In view of these facts we think it cannot be questioned, that few diseases are more deserving of attention than rheumatism; and next to its treatment no point connected with the malady would seem to have higher interest than its cause. Almost universally since the affection first attracted the attention of medical men it has been referred to cold, and yet the analogies between rheumatism and gout, remarked by Sydenham, ought to have excited suspicion long ago that the cause of the disorder might be one generated within the system rather than one acting from without. The author of the work before us holds that rheumatism has its origin in "a vitiated condition of the circulating fluids," and in favor of that opinion he refers to the more prominent features of the disease, its attacks ushered in by premonitory fever, its local symptoms erratic, yet remarkably symmetrical, and the very common consequence of affections of the heart, lungs, and other internal organs. The materies morbi, he inclines with Dr. Prout and Dr. Todd to believe, is *lactic acid*, the product of mal-assimilation, which accumulates in consequence of defective cutaneous action.

This being the pathology of rheumatism, according to Dr. Fuller, the treatment suggested is one which will neutralize the acid, or eliminate it from the system, and nothing, in this view of the affection, can be more hopeless than the attempt to subdue the fever, allay the pain in the joints, or control the other symptoms of the disease, so long as the blood is infected by the poison. The great danger of the complaint being inflammation of the heart, an important indication is to avoid anything likely to excite or irritate that organ. We give the author's plan of cure in his own words:

"The treatment to which I usually have recourse at the outset of the attack, is that alluded to at the beginning of this chapter. It is made up of alkalies and the neutral salts, with colchicum, calomel, and opium. Sometimes a little antimony is added, sometimes the aid of purgatives is had recourse to, and occasionally, though rarely, I deem it expedient to premise a moderate blood-letting. Baths are never employed if the skin is acting freely; but if, instead of being bathed in perspiration, it remains dry and hot, and burning, I then endeavor to stimulate its action by means of the vapor or the hot air bath.

"As venesection, if employed at all in acute rheumatism, is to be made use of with the view of producing an impression on the train of morbid actions, and expediting the operation of other remedies, it must necessarily take the lead of all other measures; and the first question to be decided, therefore, in every case of acute rheumatism, is as to the propriety of having recourse to its employment. It has been already pointed out, that the use of the lancet is not necessary for the relief of the pain or the tranquilization of the pulse, and that in the pale and weakly it exercises an influence prejudicial to the patient by rendering more irritable his already irritable and excited heart. But in the young, plethoric, and robust, in whom secretion is insufficient, whose pulse is full and bounding, and whose skin is dry and hot, and burning, it certainly does assist in expediting the action of other remedies, and so in promoting recovery. These, therefore, are the only cases in which it should be employed, and a single bleeding of from eight to ten ounces is generally sufficient. It relieves that excessive congestion on which the want of secretion, in great measure, depends, and which forms an obstacle to the action of those remedies on which we rely for effecting a cure.

"The next point is as to the expediency of giving calomel and purgatives. If the bowels are acting once a day, it is seldom necessary to make a more frequent call upon their activity, but a dose of calomel and opium may be prescribed with the view of modifying the character of their secretions. If the bowels are sluggish in their action, and the dejections dark colored and offensive, a dose of calomel combined with opium should be administered at once, and followed after the lapse of six or eight hours, by a draught containing an infusion of senna, together with half an ounce of the potassio tartrate of soda, and twenty minims of the vinum colchici. And the amount of opium should be so adjusted to the dose of the purgative, as to procure one full and copious evacuation without the distress attendant upon purging.

When once the bowels have been freely acted on, the state of the secretions must be our guide as to the repetition of the calomel and the morning laxative. If the tongue be rather dry; if the bowels continue sluggish, and the dejections dark colored and offensive, the mercurial and the purgative should both be repeated for several successive days. If, on the other hand, secretions from the bowels be healthy, the further exhibition of mercury is unnecessary. If, again, the secretions be copious but unhealthy in character, the calomel and opium should be repeated at night, but need not be followed by a purgative in the morning, as after one or two doses of the mercurial, the motions become lighter colored, more bilious in appearance, and of a less offensive character.

"Whilst the state of the intestinal secretions are thus attended to, alkalies or the neutral salts should be administered in combination with colchicum, full doses of opium, and sometimes a little antimony. At one time I used to content myself by giving a saline draught, with the addition of fifteen or twenty grains of the carbonate of potash, or the carbonate of soda, three or four times in the course of the day, but it soon became apparent that in order to obtain the full benefit of alkalies, it is necessary to give them in very much larger quantities—in doses proportioned to the extreme acidity of the system.* In large but ordinary doses they generally mitigated the severity of the symptoms, yet failed in affording more than partial relief, but when they were exhibited in sufficient quantities, and in combination with other reme-

---

* Though I speak throughout of the administration of alkalies, I generally prescribe the potassio tartrate of soda, which, readily undergoing decomposition in the stomach, acts quite as energetically as corresponding quantities of the alkaline carbonates, and is much more readily tolerated by the stomach.

dies, the most agonizing pain was speedily removed, and the fever subdued with marvellous rapidity. I have, therefore, ever since administered them largely, and have pushed them until my object has been attained. Nor have I seen reason, on any one occasion, to hesitate in following out this plan of treatment. It has now been pursued in a large number of cases, and in almost every instance has produced the most astonishingly favorable results. The patients have speedily lost their pains and have proceeded to convalescence. In twenty-three out of thirty-nine cases in my note-book, the pulse was tranquilized within forty-eight hours from the commencement of treatment, and in twenty-eight the pain was lulled, and the local inflamation greatly subdued within the same time, whilst in the remaining cases a longer period was required, in consequence either of previous constipation, or of the co-existence of some internal cómplication.

The form in which I usually administer the remedies, is that of a simple saline or a nitre draught, to which, if the patient be a person of average strength and robustness, bathed in profuse perspiration, with red, swollen, and exquisitely painful joints, a furred tongue, loaded with urine, and a full and bounding pulse, I usually add from two to three drachms of the potassio tartrate of soda,* ten or fifteen minims of the vinum colchici, from fifteen to twenty minims of the vinum antimonii, and from ten to fifteen minims of the tinctura opii, or of Battley's sedative solution, to prevent the salt running off by the bowels. This draught is repeated, for the first twelve or twenty-four hours, at intervals of three or four hours, according to the strength of the patient and the severity of the attack; and if the pain is excessive, I prescribe a pill containing from half a grain to a grain and a half of opium, or an equivalent dose of Dover's powder to be taken once or twice a day, taking care to increase or diminish the quantity of the sedative, according to the circumstances of the case; on the one

---

* The virtue of alkalies and their salts in rheumatism depends, I believe, upon their power.

1st. Of acting as restorers of all the alkaline condition of the system.

2dly. Of assisting to maintain the solubility of the fibrin, and thereby preventing its deposition on the valvular apparatus of the heart.

3dly. Of acting most powerfully as sedatives, and calming the action of the heart and arteries.

4thly. Of increasing the metamorphosis of tissue, and proving active provocatives of an increased secretion of urine, whereby the elimination of the materies morbi is assisted.

Whatever their mode of action, however, their effect in restoring the alkalinity of the system, and of allaying the fever and subduing the pain and inflamation, which accompany, if they be not consequent upon, the opposite condition, is very remarkable, as is also their influence in calming the action of the heart and arteries, and in causing an increased flow of urine, and a vast augmentation in its solid constituents. These facts receive very striking illustration in the cases reported at the end of this chapter.

hand, avoiding constipation and narcotism, and on the other, guarding against diarrhœa.

"Sometimes, though rarely, the stomach does not easily tolerate these large doses of the neutral salts, and in such cases, the greatest benefit is derived from the addition of a little lemon juice and an alkaline carbonate, forming a saline effervescing draught. With this variation an instance rarely occurs in which the medicine deranges the stomach, or produces the slightest disagreeable effects.

When once the medicine has begun to take effect, which is evidenced by the gradual decrease of the pain, the tranquilization of the pulse, and the increase in the quantity and specific gravity of the urine; it is repeated every fourth hour only, and then every fifth or sixth hour; and usually at the expiration of two or three days, I find its work in great measure accomplished: the saliva, by that time has lost its acidity, the pains and inflamation have subsided; the pulse has fallen, probably, from 120 to 85 or 90 beats in a minute; the tongue has become moister and less red and furred; the urine more abundant, less loaded with the lithates, and of a higher specific gravity; and the perspiration less acid, less sour smelling, and less profuse. In proportion as these symptoms of amendment manifest themselves, so is the dose of the alkalies decreased, until after the lapse of three or four days, I usually feel justified in commencing the administration of quina during the day, taking care to maintain a free action of the bowels by exhibiting, now and then at bedtime, two or three grains of the acetous extract of colchicum, together with aloes or rhubarb, and if necessary, a grain of calomel or blue pill. Should there be the slightest return of pain, the least increase of coating on the tongue, or, indeed, any evidence of returning fever, the use of quina is at once abandoned, and alkalies are again resorted to."

We regard this monograph of Dr. Fuller's as a valuable contribution to our literature on practical medicine. It is an exceedingly interesting work, and we cordially recommend it to our readers, as one from which they will derive much sound philosophy and much reliable practice.

